# How COVID-19 Impacted Older Adult Walking Group Members in Scotland: A Mixed-Methods Study

**DOI:** 10.1093/geroni/igab046.1774

**Published:** 2021-12-17

**Authors:** Kathryn Martin, Kevin Stelfox, Wei Lynn Khor, Amudha Poobalan, Daniel Powell, Rute Vieira, Marjory D'Arcy, Peter Murchie

**Affiliations:** 1 University of Aberdeen, Aberdeen, Scotland, United Kingdom; 2 Grampian; 50 + Network, Aberdeen, Scotland, United Kingdom

## Abstract

Scotland has enacted strict social distancing and stay-at-home policies during the COVID-19 pandemic, at times prohibiting outdoor group-based physical activity. This mixed-method study examined the changing role of older adult walking groups in North East Scotland around the first lockdown and how restrictions impacted members’ well-being. Three consecutive surveys were posted or emailed to members of the Grampian 50+ Network over summer 2020, with questions about social contact, loneliness, well-being, physical activity, public health messages, help-seeking behavior, and socio-demographics. 346 members completed the June survey, with 268 (83%) returning the follow-up survey in July, and 258 (80%) in August. Twenty participants (selection criteria - gender and geographic location) participated in repeated semi-structured interviews. Participants were, on average, 72±7 years old (range: 58-90), retired (94%), and women (80%). Participants reported missing in-person interaction from not regularly meeting with their walking group. Groups adapted to stay-at-home measures by using technology (i.e. videoconferencing/text/email/telephone) to maintain relationships. Easing restrictions required groups to modify format, location and size. Concerns about safe transport, mask-wearing, maintaining social distance (2m/6ft), and potential lack of socialisation emerged as barriers for future engagement. While, participants generally expressed confidence in the Scottish Government’s pandemic response and public health messaging, they expressed dissatisfaction that ‘over-70s’ were grouped together. Findings suggest that these walking group members fared well and were adaptive in response to the pandemic. Promoting group-based opportunities for physical activity and social interaction remain vital for the health and well-being of older adults in the near and long term.

